# Tick borne encephalitis without cerebrospinal fluid pleocytosis

**DOI:** 10.1186/s12879-014-0614-0

**Published:** 2014-11-18

**Authors:** Daša Stupica, Franc Strle, Tatjana Avšič-Županc, Mateja Logar, Blaž Pečavar, Fajko F Bajrović

**Affiliations:** Department of Infectious Diseases, University Medical Center Ljubljana, Japljeva 2, Ljubljana, 1525 Slovenia; Institute of Microbiology and Immunology, Faculty of Medicine Ljubljana, Ljubljana, Slovenia; Institute of Pathophysiology, Faculty of Medicine Ljubljana, Zaloška 4, Ljubljana, 2211 Slovenia; Department of Neurology, University Medical Center Ljubljana, Ljubljana, Slovenia

## Abstract

**Background:**

Tick borne encephalitis is the most frequent vector-transmitted infectious disease of the central nervous system in Europe and Asia. The disease caused by European subtype of tick borne encephalitis virus has typically a biphasic clinical course with the second phase presenting as meningitis, meningoencephalitis, or meningoencephalomyelitis. Cerebrospinal fluid pleocytosis is considered a condition sine qua non for the diagnosis of neurologic involvement in tick borne encephalitis, which in routine clinical practice is confirmed by demonstration of serum IgM and IgG antibodies to tick borne encephalitis virus.

**Case presentation:**

Here we present a patient from Slovenia, an area highly endemic for tick borne encephalitis, with encephalitis but without cerebrospinal fluid pleocytosis in whom tick borne encephalitis virus infection of the central nervous system was demonstrated.

**Conclusion:**

Cerebrospinal fluid pleocytosis is not mandatory in encephalitis caused by tick borne encephalitis virus. In daily clinical practice, in patients with neurologic symptoms/signs compatible with tick borne encephalitis and the risk of exposure to ticks in a tick borne encephalitis endemic region, the search for central nervous system infection with tick borne encephalitis virus is warranted despite the lack of cerebrospinal fluid pleocytosis.

## Background

Tick borne encephalitis (TBE) is the most frequent vector-transmitted infectious disease of the central nervous system (CNS) in Europe and Asia and is considered an emerging disease due to its rising incidence and the spread of endemic areas in recent decades [1]. TBE caused by European subtype of TBE virus (TBEV) has typically a biphasic clinical course with the second phase presenting as meningitis, meningoencephalitis, or meningoencephalomyelitis [2]. Cerebrospinal fluid (CSF) pleocytosis is considered a condition sine qua non for the diagnosis of CNS involvement in TBE, which in routine clinical practice is confirmed by demonstration of serum IgM and IgG antibodies to TBEV [2]-[4]. Cases of TBE with neurologic involvement but without CSF pleocytosis have been published [5]-[7], however, only the case recently reported by Pöschl et al. was convincingly substantiated [5]. Here we present a patient from Slovenia, an area highly endemic for TBE [8], with clinical features of encephalitis, who fulfilled criteria for recent CNS infection with TBEV although he had no CSF pleocytosis.

## Case presentation

A 79-year-old man with arterial hypertension and chronic venous ulcers on both shins, fell ill acutely with diarrhea, fatigue and sleepiness in midsummer 2013. After a week, diarrhea stopped, but he became febrile up to 38.5°C and was no longer able to walk independently due to general weakness. As a beekeeper he had been exposed to ticks in the past but could not remember having had a tick bite during the preceding few months. At admission to hospital on day 8 of his illness, he was lethargic, disoriented, but without signs of meningeal irritation. His blood pressure was 133/83 mmHg, heart rate 99/min, breathing rate 30/min and axillary temperature 38.9°C. Routine laboratory blood tests revealed normal blood cell count, mild hyponatremia (Na 129; normal 135-145 mmol/l), and slightly elevated concentrations of C-reactive protein (32 mg/l; normal 0-5 mg/l), liver enzymes (aspartate aminotransferase 0.73; normal ≤0.58 μkat/l, gamma-glutamyl transpeptidase 1.12 μkat/l; normal ≤0.92 μkat/l) and creatinine (101 μmol/l; normal 44-97 μmol/l). CSF examination yielded elevated protein concentration (1.31 g/l; normal 0.15-0.45 g/l), but normal leukocyte count (3 × 10^6^/l; normal ≤5 × 10^6^/l) and glucose concentration. In the following days the patient remained febrile up to 39.4°C. On day 10, tremor of hands and tongue appeared and his mental status deteriorated to somnolence. Computed tomography of the brain showed only mild periventricular leukopathy. Repeated CSF analyses on day 14 and 23 revealed elevated protein concentrations (1.23, and 2.02 g/l, respectively), but still no pleocytosis (CSF leukocyte count 1, and 2 × 10^6^/l, respectively). PCR analyses of CSF for the presence of TBEV on day 8 and 23 were negative as were for HSV 1, HSV 2, VZV, and enteroviruses. Based on serological results the patient did not have Lyme neuroborreliosis. However, serum IgM and IgG antibodies to TBEV were demonstrated using enzyme linked immunosorbent assay - ELISA (Enzygnost Anti-TBE/FSME Virus IgG, IgM; Siemens, Marburg, Germany) (Table 1). The follow-up levels of specific serum antibodies and the avidity of specific serum IgG (12.7%, 15.4%, and 51.6% on day 14, 21, and 65, respectively) indicated recent infection with TBEV. In addition, demonstration of intrathecal production of anti-TBEV IgM and IgG verified CNS infection with TBEV (Table 1). From day 14 the patient was no longer febrile and his mental and physical status progressively improved. Hospitalization was prolonged because of hospital acquired pneumonia which was treated with amoxicillin/clavulanate. At transfer to a nursing facility on day 32 the patient was afebrile, completely oriented, feeble, but without focal neurological deficit. Routine laboratory test results were unremarkable.

**Table 1:** **Enzyme linked immunosorbent assay findings**

	Anti-TBEV				IgM antibody index ^a^	IgG antibody index ^a^
	IgM in serum	IgG in serum	IgM in CSF	IgG in CSF		
Day 14	1.473	78.4	0.601	53.7	NA	NA
Day 23	1.133	130	0.676	89.7	48.6	40.2

TBEV, tick-borne encephalitis virus; CSF, cerebrospinal fluid; NA, not available.

^a^Antibody index of intrathecal synthesis of anti-TBEV IgM and IgG calculated according to Reiber’s formula [9].

## Conclusions

Presenting symptoms and signs of TBE are nonspecific and similar as in acute aseptic meningoencephalitis of other etiologies with fever in 92%, vomiting in 38%, headache in 67-100%, altered consciousness in 12-35.5%, seizures in 0.3-3.3%, tremor in 7-78%, dysphasia in 0.7-3.8%, spinal nerve paralysis in 2.7-15%, and cranial nerve paralysis in 3.3-11% of cases [2],[10]. Accordingly, our patient with fever, altered consciousness and tremor of the hands and tongue had clinical indications of acute encephalitis.

In our patient, recent infection with TBEV was demonstrated by the presence of specific IgM and IgG antibodies in serum and further substantiated with the appropriate increase in antibody levels and IgG avidity in convalescent samples. Since detailed history revealed that he had not had TBE in the past, had not traveled outside Slovenia, nor was he vaccinated against TBE, yellow fever or Japanese encephalitis, and he had negative serum antibodies against West Nile virus, the only potential coinfecting flavivirus in Slovenia, the possibility of false positive serological results for TBE due to cross-reaction is highly improbable. In routine clinical practice the finding of specific IgM and IgG serum antibodies in a patient residing in endemic area, who has clinical signs/symptoms of meningitis/encephalitis associated with elevated CSF leukocyte count, is considered confirmatory for TBE [2],[3]. However, demonstration of recent infection with TBEV by itself does not attest for CNS involvement; in fact the majority of infections with TBEV are asymptomatic or without prominent morbidity [2]. For a reliable diagnosis of unusual TBE cases such as ours without CSF pleocytosis, which is most likely rather rare and therefore associated with low pre-test probability, not only evidence of a recent infection, but also evidence of CNS infection with TBEV is needed. We were unable to demonstrate the presence of TBEV in CSF by PCR, which is not surprising in regard to earlier reports that the virus can be detected by PCR in blood during the initial phase of TBE, but extremely rarely in blood or CSF during the second phase of the disease [11]. However, we ascertained CNS infection by demonstration of intrathecal synthesis of specific antibodies to TBEV, which is a much more sensitive approach for verifying CNS infection. The main source of CSF antibodies are lymphocytes B, but in patients with TBE lymphocytes B comprise only 3-5% of CSF lymphocyte population [12]. Therefore, one may hypothesize, that the major source of CSF antibodies are lymphocytes B accumulated in the perivascular spaces of brain parenchyma [13].

In reports including large number of patients with proven TBE, CSF pleocytosis was established in all cases [2],[4],[10],[14]. This finding, however, might be due to a selection bias because in these studies CSF pleocytosis was one of the inclusion criteria for the diagnosis of TBE [15]. Our case, together with the recently published case by Pöschl et al. [5], and some earlier less convincing reports [6],[7], show that CSF pleocytosis is not mandatory in patients with encephalitis caused by TBEV. This is in accordance with the reports on encephalitis caused by some other viruses such as herpes simplex 1 in which about 3% of cases take place with normal CSF leukocyte count [16]. The frequency of encephalitis without CSF pleocytosis caused by TBEV infection remains to be determined. However, the paucity of reports implies that such presentation is most probably very rare.

The findings in our patient suggest that in daily clinical practice, in patients with symptoms/signs of encephalitis compatible with TBE and the risk of exposure to ticks in a TBE endemic region, the search for infection with TBEV is warranted despite the lack of CSF pleocytosis. However, to reliably prove that the encephalitis with normal CSF white cell count is caused by TBEV, a proof of TBEV CNS infection is needed besides the usual diagnostic requirements i.e., the evidence of a recent infection with TBEV denoted by the presence of specific serum IgM and IgG antibodies.

## Consent

Written informed consent was obtained from the patient for publication of this Case report. A copy of the written consent is available for review by the Editor of this journal.
